# A powerful parent-of-origin effects test for qualitative traits on X chromosome in general pedigrees

**DOI:** 10.1186/s12859-017-2001-5

**Published:** 2018-01-05

**Authors:** Qi-Lei Zou, Xiao-Ping You, Jian-Long Li, Wing Kam Fung, Ji-Yuan Zhou

**Affiliations:** 1 State Key Laboratory of Organ Failure Research, Ministry of Education, and Guangdong Provincial Key Laboratory of Tropical Disease Research, Department of Biostatistics, School of Public Health, Southern Medical University, No. 1023, South Shatai Road, Baiyun District, Guangzhou, 510515 China; 2 Department of Statistics and Actuarial Science, University of Hong Kong, Pokfulam Road, Hong Kong, China

**Keywords:** Parent-of-origin effects, Imprinting effects, Parental-asymmetry test, X chromosome, Qualitative trait, Pedigree

## Abstract

**Background:**

Genomic imprinting is one of the well-known epigenetic factors causing the association between traits and genes, and has generally been examined by detecting parent-of-origin effects of alleles. A lot of methods have been proposed to test for parent-of-origin effects on autosomes based on nuclear families and general pedigrees. Although these parent-of-origin effects tests on autosomes have been available for more than 15 years, there has been no statistical test developed to test for parent-of-origin effects on X chromosome, until the parental-asymmetry test on X chromosome (XPAT) and its extensions were recently proposed. However, these methods on X chromosome are only applicable to nuclear families and thus are not suitable for general pedigrees.

**Results:**

In this article, we propose the pedigree parental-asymmetry test on X chromosome (XPPAT) statistic to test for parent-of-origin effects in the presence of association, which can accommodate general pedigrees. When there are missing genotypes in some pedigrees, we further develop the Monte Carlo pedigree parental-asymmetry test on X chromosome (XMCPPAT) to test for parent-of-origin effects, by inferring the missing genotypes given the observed genotypes based on a Monte Carlo estimation. An extensive simulation study has been carried out to investigate the type I error rates and the powers of the proposed tests. Our simulation results show that the proposed methods control the size well under the null hypothesis of no parent-of-origin effects. Moreover, XMCPPAT substantially outperforms the existing tests and has a much higher power than XPPAT which only uses complete nuclear families (with both parents) from pedigrees. We also apply the proposed methods to analyze rheumatoid arthritis data for their practical use.

**Conclusions:**

The proposed XPPAT and XMCPPAT test statistics are valid and powerful in detecting parent-of-origin effects on X chromosome for qualitative traits based on general pedigrees and thus are recommended.

**Electronic supplementary material:**

The online version of this article (10.1186/s12859-017-2001-5) contains supplementary material, which is available to authorized users.

## Background

Genomic imprinting is one of the well-known epigenetic factors causing the association between traits and genes, where the expression level of a gene depends on its parental origin. Imprints are laid down in the parental germ cells, which affect embryonic growth in the womb and behavior after birth [1]. Aberrant imprinting on autosomes disturbs development and consequently results in various disease syndromes, such as Beckwith-Wiedemann, Prader-Willi and Angelman syndromes [1–4]. On the other hand, the imprinted genes on X chromosome may play a substantial role in Turner’s syndrome and autism [5, 6].

Therefore, taking information on imprinting effects into account when conducting association analysis could improve the test power [7]. On the other hand, genomic imprinting has been generally examined through testing for parent-of-origin effects of alleles [8]. A lot of methods have been proposed to test for parent-of-origin effects on autosomes. For a diallelic single nucleotide polymorphism (SNP) locus and qualitative traits, the parental-asymmetry test (PAT) was proposed to test for parent-of-origin effects based on nuclear families with both parents and one affected child [9]. Then its extensions (1-PAT and C-PAT) could handle the situation with missing parental genotypes and more than one affected child [10]. For quantitative traits, He et al. [11] developed several PAT-type parent-of-origin effects tests for such a task. However, these methods are only applicable to nuclear family data. As such, Zhou et al. [12] developed the pedigree parental-asymmetry test (PPAT) for qualitative traits, which can use all available information from extended pedigrees, leading to power improvement. He et al. [13] extended PPAT to accommodate quantitative traits. On the other hand, although these parent-of-origin effects tests on autosomes have been available for more than 15 years, there has been no statistical test developed to test for parent-of-origin effects on X chromosome, until recently Zhou et al. [14] proposed the parental-asymmetry test on X chromosome (XPAT) and its extensions, which can be used to detect parent-of-origin effects on X chromosome for qualitative traits. For quantitative traits on X chromosome, Yu et al. [15] developed the Q-XPAT method to test for parent-of-origin effects. However, these methods on X chromosome are only suitable for nuclear families and thus do not accommodate general pedigrees.

In this article, inspired by the need to utilize all available family trios in a general pedigree like PPAT and to consider X chromosome as well, we propose the pedigree parental-asymmetry test on X chromosome (XPPAT) statistic to test for parent-of-origin effects in the presence of association for qualitative traits. When there are missing genotypes in some pedigrees, we further develop the Monte Carlo pedigree parental-asymmetry test on X chromosome (XMCPPAT) by inferring the missing genotypes given the observed genotypes based on a Monte Carlo estimation [12, 16], to test for parent-of-origin effects. We have carried out an extensive simulation study to investigate the type I error rates and the powers of the proposed tests. Simulation results show that the proposed methods control the size well under the null hypothesis of no parent-of-origin effects. Moreover, XMCPPAT substantially outperforms the existing tests and has a much higher power than XPPAT which only uses complete nuclear families (with both parents) from pedigrees. We also apply the proposed methods to analyze rheumatoid arthritis data for their practical use.

## Methods

### Notations

For a candidate diallelic SNP locus on X chromosome, suppose that there are two alleles, the deleterious allele *D* and the normal allele *d*, with frequencies *p* and 1−*p*, respectively, where we assume that the frequencies of the same allele in males and females are equal. Next, the females are typed into four possible genotypes *D*/*D*, *D*/*d*, *d*/*D* and *d*/*d*, where the left allele of the slash is paternal and the right one is maternal. Let *ρ* be the inbreeding coefficient in females. Then, the frequencies of genotypes *D*/*D*, *D*/*d*, *d*/*D* and *d*/*d* in females are Pr(*D*/*D*)=*p*^2^+*ρpq*, Pr(*D*/*d*)=Pr(*d*/*D*)=*pq*(1−*ρ*), and Pr(*d*/*d*)=*q*^2^+*ρpq*, respectively. When *ρ*=0, the Hardy-Weinberg equilibrium (HWE) holds in females. Also, let *f*_11_, *f*_10_, *f*_01_ and *f*_00_ be the four penetrances corresponding to genotypes *D*/*D*, *D*/*d*, *d*/*D* and *d*/*d*, respectively. Suppose that *I*=(*f*_10_−*f*_01_)/2, which is used to measure the degree of parent-of-origin effects. *I*=0 indicates no parent-of-origin effects. Note that males have only one X chromosome. So, they are not informative when we calculate the test statistics for testing parent-of-origin effects. Therefore, we define an informative family, which has at least one affected heterozygous daughter together with her parents. Further, in this article, we assume that there is no maternally-mediated effect.

A general pedigree consists of multiple two-generation nuclear families. For each nuclear family, we divide it into multiple parents-child trios, each with a child and his/her parents. However, only the trios with an affected heterozygous daughter and her parents are informative for parent-of-origin effects. For convenience, in each informative trio, let *F*, *M* and *C* denote the count of allele *D* in the father, the mother and the affected daughter, respectively. Note that there are only four genetically possible types of informative family trios *FMC*: 101, 111, 011 and 021.

### XPPAT for general pedigree data

Suppose that we collect *N* independent pedigrees, and there are *n*_*i*_ family trios in pedigree *i*,*i*=1,...,*N*. For trio *j* in pedigree *i*, let

$$R_{ij} = I_{F_{ij}\geq M_{ij}, C_{ij}=1} - I_{F_{ij}< M_{i}, C_{ij}=1}, $$

*i*=1,…,*N*;*j*=1,…,*n*_*i*_, where *I*_{comparison statement}_ is 1 when the “comparison statement” is true and 0 otherwise; *F*_*ij*_,*M*_*ij*_ and *C*_*ij*_ are the counts of allele *D* of the father, the mother and the affected daughter in trio *j* of pedigree *i*, respectively. Note that $I_{F_{ij}\geq M_{ij}, C_{ij}=1}$ indicates the copies of allele *D* in father are more than or equal to those in mother and their daughter is heterozygous, which means that the allele *D* in the daughter is paternal (*F*_*ij*_*M*_*ij*_*C*_*ij*_=101 or 111), and vice versa for $I_{F_{ij}<M_{ij}, C_{ij}=1}$ (*F*_*ij*_*M*_*ij*_*C*_*ij*_=011 or 021). Therefore, $S_{i} = \sum _{j=1}^{n_{i}}R_{ij}$ will provide the information on parent-of-origin effects. Under the null hypothesis of no parent-of-origin effects, from Zhou et al. [14], we have E(*S*_*i*_)=0 and $\mathrm {E}\left (\sum _{i=1}^{N}S_{i}\right)=0$. So,

$$\begin{aligned} {\text{Var} \left(\sum\limits_{i=1}^{N}S_{i} \right)} &= \sum\limits_{i=1}^{N}{\text{Var}}\left(S_{i}\right) = \sum\limits_{i=1}^{N}{\mathrm{E}}\left(S_{i}^{2}\right)\\ &= {\mathrm{E}}\left(\sum\limits_{i=1}^{N}S_{i}^{2}\right)\\ &= {\mathrm{E}}\left[\sum\limits_{i=1}^{N}\left (\sum\limits_{j=1}^{n_{i}}\emph R_{ij}\right)^{2}\right]. \end{aligned} $$

Therefore, $\sum _{i=1}^{N}\left (\sum _{j=1}^{n_{i}} R_{ij}\right)^{2}$ is an unbiased estimate of the variance of $ \sum _{i=1}^{N} S_{i}$.

Then we construct the following XPPAT test statistic for general pedigrees to test for parent-of-origin effects on X chromosome:

1 $$\begin{array}{@{}rcl@{}} \text{XPPAT} &= \frac{\sum \limits_{i=1}^{N}S_{i}}{\sqrt{\sum \limits_{i=1}^{N}S_{i}^{2}}}= \frac{\sum \limits_{i=1}^{N}\sum \limits_{j=1}^{n_{i}} R_{ij}}{ \sqrt{\sum \limits_{i=1}^{N}\left (\sum \limits_{j=1}^{n_{i}}R_{ij}\right)^{2}}}. \end{array} $$

When the number of pedigrees is large enough, XPPAT follows a standard normal distribution approximately.

### XMCPPAT when the genotypes of some individuals are missing

When there are missing genotypes for some individuals in some pedigrees, XPPAT only uses the informative family trios without missing genotypes from each pedigree, and simply ignores other family trios with missing data, which may cause the loss in power. Thus, to improve the test power, we extend XPPAT to XMCPPAT which can handle this situation. Specifically, a Monte Carlo (MC) sampling procedure is used to infer the missing genotypes *G*_*m*_ given the observed genotypes *G*_*o*_ in each pedigree. Let *S* be the contribution of a pedigree to the statistic XPPAT in Eq. (1), and *S*_*MC*_ denotes the conditional expectation of *S* given the observed genotypes *G*_*o*_. Here, for simplicity, the subscripts are suppressed without causing ambiguity. So,

2 $$\begin{array}{@{}rcl@{}} S_{MC} =\mathrm{E}[S | G_{o}] = \mathrm{E}[S(G_{m}, G_{o}, A) | G_{o}], \end{array} $$

where *S*(*G*_*m*_,*G*_*o*_,*A*) depends on the missing genotypes (*G*_*m*_), the observed genotypes (*G*_*o*_) and the collection of the observed phenotypes of all the individuals in the pedigree (*A*). Note that to calculate *S*_*MC*_, it is computationally intensive and time consuming due to the huge amounts over all possible missing genotypes *G*_*m*_ given *G*_*o*_. So, we follow Zhou et al. [12] and Ding et al. [16] by taking the following MC simulation scheme to estimate *S*_*MC*_. Firstly, we generate *K* independent samples *G*_*mk*_,*k*=1,…,*K* from Pr(*G*_*m*_|*G*_*o*_) by using the SLINK software based on the peeling algorithm of Weeks et al. [17]. Then, take the arithmetic mean of all the *S*(*G*_*mk*_,*G*_*o*_,*A*)’s as the estimate of *S*_*MC*_,

$$\begin{array}{@{}rcl@{}} S_{MC} \approx \frac {1}{K}\sum \limits_{k=1}^{K}S(G_{mk}, G_{o}, A).  \end{array} $$

To this end, we calculate the statistic XPPAT in Eq. (1) by replacing each *S* by *S*_*MC*_ and obtain the following XMCPPAT test

$$\begin{array}{@{}rcl@{}} \text{XMCPPAT} &= \frac{\sum \limits_{i=1}^{N}S_{MCi}}{\sqrt{\sum \limits_{i=1}^{N}S_{MCi}^{2}}}. \end{array} $$

Under the null hypothesis of no parent-of-origin effects, we have E(*S*_*MC*_)=0 [see Appendix A of Additional file 1]. Note that Pr(*G*_*m*_|*G*_*o*_) may be different from Pr(*G*_*m*_|*G*_*o*_,*A*). So, we treat *A* as random and the minimal ascertainment criterion used is that only pedigrees with at least one affected daughter can be included, just like Zhou et al. [12] and Ding et al. [16].

### Simulation settings

**Fig. 1:**
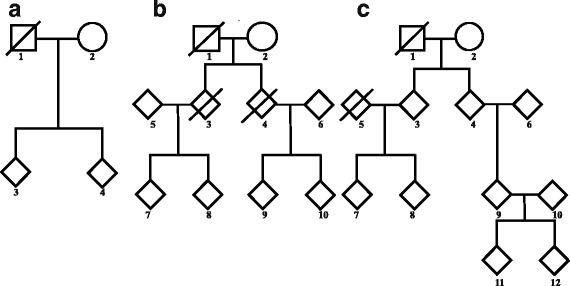
Three pedigree structures used for the simulation study. The (**a**), (**b**) and (**c**) parts represent two-, three- and four-generation pedigrees, respectively. The squares are males, and the circles are females. The rhombus could be any gender. **“/”** denotes that the genotype of the individual is missing

To evaluate the performance of the proposed XPPAT and its extension XMCPPAT, we conduct a simulation study to compare them with the existing XPAT. We consider three different pedigree structures respectively including two, three and four generations as shown in Fig. 1. Note that the squares and the circles indicate male founders and female founders in the first generations, respectively. Meanwhile, all the nonfounders as well as their heterosexual mates are represented by rhombuses, which means that the gender of each nonfounder could be male or female. The sexual proportion is fixed at 1:1 in our simulation study. When a person has “/” on his or her pattern, his or her genotype is set to be missing. For example, the genotypes of the first, third and fourth members of the three-generation pedigree in Fig. 1b are missing. The number *N* of pedigrees is taken as 150 and 300 with the ratio of the three structures being 1:1:1.

The frequency *p* of allele *D* is fixed to be 0.1 and 0.3. The inbreeding coefficient *ρ* in females is taken as -0.05, 0 and 0.05. We choose five parent-of-origin effect models: (*f*_11_,*f*_10_,*f*_01_,*f*_00_)=(0.30,0.21,0.21,0.12) with *f*_10_=*f*_01_ being assigned for simulating the type I error rates of the proposed tests, while S1: (*f*_11_,*f*_10_,*f*_01_,*f*_00_)=(0.30,0.30,0.12,0.12) (complete maternal parent-of-origin effect), S2: (*f*_11_,*f*_10_,*f*_01_,*f*_00_)=(0.30,0.12,0.30,0.12) (complete paternal parent-of-origin effect), S3: (*f*_11_,*f*_10_,*f*_01_,*f*_00_)=(0.30,0.26,0.16,0.12) (incomplete maternal parent-of-origin effect) and S4: (*f*_11_,*f*_10_,*f*_01_,*f*_00_)=(0.30,0.16,0.26,0.12) (incomplete paternal parent-of-origin effect) for the power investigation.

We use the nominal significance levels *α*=5*%* and 1% for the type I error rate assessment and set *α*=5*%* for the power investigation. The simulated type I error rates and powers are based on 10,000 replications. For each replication, 50 Monte Carlo samples of missing genotypes are generated by using the SLINK software [17]. We consider the following seven test statistics (four versions of XMCPPAT, two versions of XPPAT and one version of XPAT). Note that the allele frequencies are needed in the MC sampling procedure. So, we consider the following four versions of XMCPPAT: XMCPPAT_*t*_, XMCPPAT_*f*_, XMCPPAT_*m*_ and XMCPPAT_*fm*_, which are based on the true allele frequencies, those estimated from female founders, male founders and both female and male founders, respectively. Further, XPPAT_*full*_ denotes the test for complete data without any missing data (assuming that the genotypes of individual 1 in two-generation families, individuals 1, 3 and 4 in three-generation pedigrees, and individuals 1 and 5 in four-generation pedigrees are available), which can be considered as the gold standard. XPPAT deals with pedigrees after removing missing data without using the MC procedure. That is, XPPAT only uses individuals 4, 6, 9, 10, 11 and 12 in four-generation pedigrees. As for XPAT, we use the youngest two-generation nuclear families in four-generation pedigrees having individuals 9, 10, 11 and 12.

## Results

### Type I error rates and powers

Table 1 shows the estimated type I error rates of the proposed methods against different *α* (0.05 and 0.01), *N* (150 and 300), *p* (0.1 and 0.3) and *ρ* (-0.05, 0 and 0.05) values under the null hypothesis of no parent-of-origin effects. It can be seen from the table that XPPAT_*full*_, XMCPPAT_*t*_ and XMCPPAT_*fm*_ control the type I error rate well. Most of the size results of XMCPPAT_*f*_ are quite good, except for some appearing little conservative. On the other hand, some of the type I error rates of XMCPPAT_*m*_ based on the estimated allele frequencies from male founders are inflated. So, we only conduct power comparison based on the true allele frequencies and those estimated from both female and male founders later. The size results of X*PPAT* and XPAT are also generally close to the nominal level 5% when *N*=300. However, other empirical type I error rates of XPPAT and XPAT are smaller than the respective nominal significance levels, especially for *α*=1*%*. This may be because the number of the informative families for XPPAT and XPAT is small. In addition, it appears that there is little impact of *ρ* on the validity of the proposed tests.

**Table 1:** Empirical size (%) of XPPAT_full_, XMCPPAT_t_, XMCPPAT_f_, XMCPPAT_m_, XMCPPAT_fm_, XPPAT and XPAT under the null hypothesis

*α*	*N*	*p*	*ρ*	XPPAT_full_	XMCPPAT_t_	XMCPPAT_f_	XMCPPAT_m_	XMCPPAT_fm_	XPPAT	XPAT
0.05	150	0.1	-0.05	4.73	4.64	4.28	5.20	4.80	4.30	4.39
	150	0.1	0	4.76	4.93	4.49	5.35	4.88	4.74	4.64
	150	0.1	0.05	5.09	4.87	4.52	5.50	5.12	4.53	4.68
	150	0.3	-0.05	4.67	4.66	4.10	6.27	5.00	4.44	4.52
	150	0.3	0	4.93	4.96	4.53	6.65	5.26	5.00	4.55
	150	0.3	0.05	4.91	4.99	4.38	6.12	5.19	4.57	4.17
	300	0.1	-0.05	4.76	4.89	4.70	5.62	5.08	5.00	4.69
	300	0.1	0	5.23	5.00	4.89	5.72	5.11	4.77	4.50
	300	0.1	0.05	5.12	4.90	4.62	5.55	5.05	4.48	4.78
	300	0.3	-0.05	4.93	5.33	4.76	7.05	5.45	5.16	5.16
	300	0.3	0	4.98	5.12	4.41	6.44	5.29	5.38	5.27
	300	0.3	0.05	4.93	5.07	4.95	6.71	5.63	5.43	4.99
0.01	150	0.1	-0.05	0.88	0.69	0.64	0.92	0.73	0.40	0.18
	150	0.1	0	1.01	0.86	0.75	0.99	0.88	0.31	0.19
	150	0.1	0.05	0.97	0.82	0.77	1.04	0.83	0.43	0.26
	150	0.3	-0.05	0.84	0.92	0.78	1.31	0.97	0.63	0.37
	150	0.3	0	0.99	0.84	0.74	1.45	0.99	0.90	0.81
	150	0.3	0.05	1.04	0.92	0.73	1.47	0.97	0.72	0.78
	300	0.1	-0.05	0.84	0.93	0.87	0.97	0.94	0.76	0.62
	300	0.1	0	0.98	1.00	0.97	1.21	1.02	0.75	0.88
	300	0.1	0.05	0.92	0.99	0.86	1.21	1.07	0.73	0.66
	300	0.3	-0.05	1.03	1.05	0.89	1.75	1.10	0.83	0.68
	300	0.3	0	0.96	1.02	0.85	1.51	1.08	0.93	0.83
	300	0.3	0.05	1.06	0.96	0.88	1.55	1.13	0.80	0.83

**Fig. 2:**
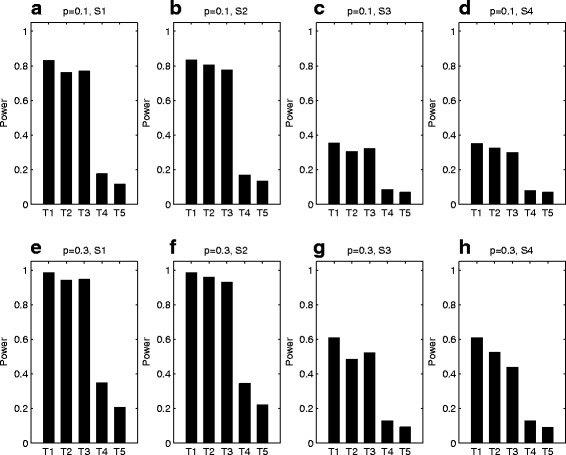
Power comparison of T1: XPPAT_*full*_, T2: XMCPPAT_*t*_, T3: XMCPPAT_*fm*_, T4: XPPAT and T5: XPAT. The powers are calculated under four different parent-of-origin effect models of S1: (*f*_11_,*f*_10_,*f*_01_,*f*_00_)=(0.30,0.30,0.12,0.12), S2: (*f*_11_,*f*_10_,*f*_01_,*f*_00_)=(0.30,0.12,0.30,0.12), S3: (*f*_11_,*f*_10_,*f*_01_,*f*_00_)=(0.30,0.26,0.16,0.12) and S4: (*f*_11_,*f*_10_,*f*_01_,*f*_00_)=(0.30,0.16,0.26,0.12) with *N*=150 and *ρ*=0 based on 10,000 replicates at the significance level of 5%. The first four tests are the proposed tests, while the last one is the existing test. The first row (**a**), (**b**), (**c**) and (**d**) with *p*=0.1, while the second row (**e**), (**f**), (**g**) and (**h**) with *p*=0.3

**Fig. 3:**
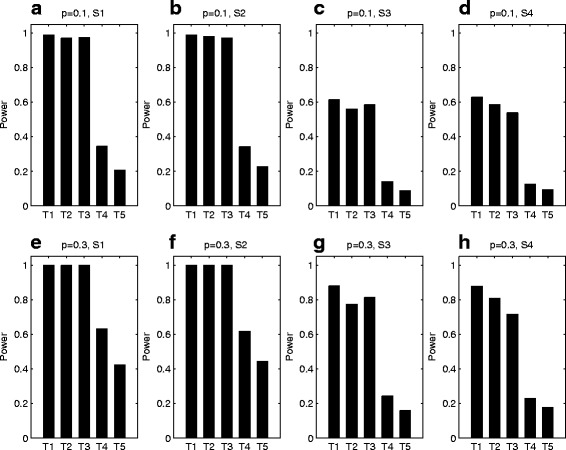
Power comparison of T1: XPPAT_*full*_, T2: XMCPPAT_*t*_, T3: XMCPPAT_*fm*_, T4: XPPAT and T5: XPAT. The powers are calculated under four different parent-of-origin effect models of S1: (*f*_11_,*f*_10_,*f*_01_,*f*_00_)=(0.30,0.30,0.12,0.12), S2: (*f*_11_,*f*_10_,*f*_01_,*f*_00_)=(0.30,0.12,0.30,0.12), S3: (*f*_11_,*f*_10_,*f*_01_,*f*_00_)=(0.30,0.26,0.16,0.12) and S4: (*f*_11_,*f*_10_,*f*_01_,*f*_00_)=(0.30,0.16,0.26,0.12) with *N*=300 and *ρ*=0 based on 10,000 replicates at the significance level of 5%. The first four tests are the proposed tests, while the last one is the existing test. The first row (**a**), (**b**), (**c**) and (**d**) with *p*=0.1, while the second row (**e**), (**f**), (**g**) and (**h**) with *p*=0.3

Figures 2 and 3 plot the estimated powers of the proposed methods and the existing XPAT test under different parent-of-origin effect models when the inbreeding coefficient *ρ* is 0, with *N*=150 and 300, respectively. The corresponding power results for *ρ*=−0.05 and 0.05 are given in Figs. A–D in Additional file 1. Note that the first four tests in all the figures are the proposed tests, while the last one is the existing test. From Figs. 2 and 3, the powers of XMCPPAT_*t*_ and XMCPPAT_*fm*_ are very close to each other, which are merely a little less than the gold standard XPPAT_*full*_. This indicates that XMCPPAT_*t*_ and XMCPPAT_*fm*_ can recapture much of missing information. Further, XMCPPAT_*t*_ and XMCPPAT_*fm*_ are much more powerful than the proposed XPPAT test and the existing XPAT test. Since the missing data are omitted, XPPAT, which only uses individuals 4, 6, 9, 10, 11 and 12 in four-generation pedigrees, suffers from substantial power loss under all the situations. However, XPPAT still has better power than XPAT, which only uses individuals 9, 10, 11 and 12 in four-generation pedigrees. The powers of all the tests under the complete parent-of-origin effect models (S1 and S2) are much higher than those under the incomplete models (S3 and S4). When the frequency *p* of allele *D* increases from 0.1 to 0.3 and *ρ* is fixed, the powers of the proposed tests are higher as the bars in the second row of both figures are taller than those in the first row. This is mainly because the number of affected heterozygous daughters will be larger as the frequency *p* increasing, which means that the number of the collected informative trios under *p*=0.3 is bigger than that under *p*=0.1. By comparing Fig. 2 with Fig. 3, we find that the powers with *N*=300 are much larger than those with *N*=150. Finally, by comparing Fig. 2 with Figs. A and C, we also find that the inbreeding coefficient *ρ* has little effect on the parent-of-origin effects testing when *N*=150, similar to *N*=300 by comparing Fig. 3 with Figs. B and D [see Additional file 1].

### Application to rheumatoid arthritis data

We apply the proposed methods to a rheumatoid arthritis (RA) data set, which is made available from North American Rheumatoid Arthritis Consortium of Genetic Analysis Workshop 15 [18]. There are 293 SNP markers on X chromosome and 757 pedigrees with 8017 individuals, including 3797 males and 4220 females in this data set. Earlier researchers have found that some SNPs on X chromosome are possibly associated with the risk of developing RA [19]. Therefore, we wonder if the associated alleles on these SNPs have parent-of-origin effects.

Before using this data set, we have the following quality control (QC) rules. All the included pedigrees at least have one affected daughter. If the genotypes of all the individuals in a pedigree are unavailable, then we delete this pedigree. The pedigrees with stepfamilies are also excluded. Further, it should be noted that too many individuals’ genotypes are missing in this data set and thus, for too large pedigrees, it may take much time to calculate the value of XMCPPAT by the Monte Carlo sampling and estimation scheme. Therefore, we exclude the pedigrees with the number of members being bigger than 27. However, after filtering the original data set by the above QC rules, there are still lots of missing genotypes in the pedigrees. Note that the pedigrees with the genotypes of more than 50% individuals missing will give large variability to the analysis. So, we delete these pedigrees. After that, we ultimately obtain 246 pedigrees with 1109 individuals, including 407 males and 702 females for analysis. On the other hand, due to the large proportion of missingness, to obtain the stable allele frequency estimates, we use all the female and male founders in the original data set to estimate the allele frequency. We conduct the XMCPDT approach [16] to test for association between genes and RA as a preliminary step because XMCPPAT is valid only when this association is present. Then, we use XMCPPAT to detect parent-of-origin effects at these associated loci on X chromosome. The MC size is set to be 50. The significance levels for the association test XMCPDT and the parent-of-origin effects test XMCPPAT are taken as 5%.

Table 2 summarizes the *p*-values of XMCPDT and XMCPPAT at 13 SNPs with *p*-values of XMCPDT being less than the 5% level. It is noticed that two SNPs have *p*-values of XMCPPAT smaller than 5%. However, after taking into account multiple testing based on Bonferroni correction for XMCPDT (*α*^′^=0.05/293=0.00017), none of the *p*-values of XMCPDT is smaller than 0.00017, and thus there is no statistically significant SNP on X chromosome for the association test XMCPDT. Note that the parent-of-origin effects test XMCPPAT is valid only in the presence of association. So, XMCPPAT could not find any statistically significant SNP.

**Table 2:** Application of XMCPDT and XMCPPAT to rheumatoid arthritis data with *p*-values of XMCPDT < 5*%*

	*p*-value	
SNP name	XMCPDT	XMCPPAT
rs2238907	0.004	0.649
rs1476468	0.036	0.592
rs916685	0.003	0.862
rs1479239	0.011	0.853
rs988431	0.013	0.893
rs1264064	0.001	0.408
rs1043034	0.007	0.439
rs763183	0.014	0.419
rs2005463	0.007	0.626
rs4462068	0.049	0.264
rs209213	0.035	0.019
rs17407	0.049	0.907
rs644345	0.030	0.027

## Discussion

In this article, we propose the novel and powerful methods, XPPAT and XMCPPAT, for testing parent-of-origin effects on X chromosome in general pedigrees for qualitative traits. Our proposed methods not only can take advantage of nuclear family data, but also can use general pedigree data. Simulation study is conducted under various simulation settings, including two sample sizes, two groups of allele frequencies, three different values of inbreeding coefficient, and five different parent-of-origin effect models. The simulation results show that the type I error rates of the proposed tests are controlled well. Moreover, the powers of the proposed tests are much higher than the existing XPAT. With the MC procedure, XMCPPAT also performs well when there are missing genotypes. Further, in the simulation study, we find that the proposed XPPAT and XMCPPAT do not depend on the assumption of HWE in females as the inbreeding coefficient almost has no effect on XPPAT and XMCPPAT. Note that, for XMCPPAT, which is suitable for missing data, we have raised four different ways to evaluate the allele frequencies: true allele frequencies, those estimated from female founders and male founders, and those estimated from both female and male founders, respectively. It appears that using the estimated allele frequencies from both female and male founders, XMCPPAT_*fm*_ has nearly the same performance as XPPAT_*full*_ based on complete data without any missing genotypes and XMCPPAT_*t*_ on the basis of the true allele frequencies. This indicates that XMCPPAT_*t*_ and XMCPPAT_*fm*_ can recapture much of missing information. As such, XMCPPAT will be practicable for real data application. However, the traits we consider in this article are restricted to be qualitative. So, our future work may be conducted for quantitative traits.

On the other hand, our current manuscript only focuses on the parent-of-origin effects test based on SNP data. However, it should be noted that RNA sequencing (RNA-seq) data convey more epigenetic information than SNP data and RNA-seq data will be more commonly available with constantly decreasing cost. Thus, the most direct way to identify imprinted genes is to directly use RNA-seq data and score the differential allelic expression depending on the parent-of-origin [20]. So, we will extend our proposed methods for parent-of-origin effects on X chromosome to accommodate RNA-seq data in future.

Besides imprinting effects, X chromosome inactivation (XCI) is another important biological mechanism on X chromosome [21]. It happens during early embryonic development in females whose paternal or maternal X chromosome is silenced to achieve dosage compensation between two sexes [22]. XCI is generally a random process where both of the paternal and maternal X chromosomes have equal chance to be inactived [23]. In this regard, XCI is easily confounded with imprinting effects. Recent studies have revealed that skewed XCI is a biological plausibility, which has been defined as a significant deviation from random XCI [24–26]. A few simulation studies demonstrate that the proposed methods are still valid for testing parent-of-origin effects under random XCI and skewed XCI [see Appendix B of Additional file 1].

Finally, it should be emphasized that it is important to make a distinction among the terms “imprinting effect”, “maternal effect” and “parent-of-origin effect” [8, 27]. Parent-of-origin effect assumes that the expression level of traits in *D*/*d* offspring is different from that in *d*/*D* offspring, which is a broader concept than an imprinting effect and can be caused by genomic imprinting or other factors. Imprinting effect is the most important form of parent-of-origin effects [27]. On the other hand, maternal effect refers to genetic contribution of a mother’s genotype to her offspring via the maternally provided environment, which is another source of parent-of-origin effects. A genome scan for quantitative trait loci affecting growth- and weight-related traits in mice illustrates that maternal effects can even mimic genomic imprinting to cause parent-of-origin effects [8]. Therefore, the XMCPPAT method proposed in this article is employed as a test for parent-of-origin effects instead of a test for imprinting effects.

## Conclusions

The proposed XPPAT and XMCPPAT test statistics are valid and powerful in detecting parent-of-origin effects on X chromosome for qualitative traits based on general pedigrees and thus are recommended.

## Additional file

Additional file 1 Appendices and Supplementary figures. **Appendix A** Proof of E(*S*_*MC*_)=0 under the null hypothesis of no parent-of-origin effects; **Appendix B** Simulation study for the validity of XPPAT when testing parent-of-origin effects under X chromosome inactivation; **Figs. A and B** Power comparison of XPPAT_*full*_, XMCPPAT_*t*_, XMCPPAT_*fm*_, XPPAT and XPAT with *N*=150 and 300, respectively. The powers are calculated under four different parent-of-origin effect models with *ρ*=−0.05 based on 10,000 replicates at the significance level of 5%; **Figs. C and D** Power comparison of XPPAT_*full*_, XMCPPAT_*t*_, XMCPPAT_*fm*_, XPPAT and XPAT with *N*=150 and 300, respectively. The powers are calculated under four different parent-of-origin effect models with *ρ*=0.05 based on 10,000 replicates at the significance level of 5%. (PDF 72 kb)

## Abbreviations

MC: Monte Carlo
PAT: The parental-asymmetry test
PPAT: The pedigree parental-asymmetry test
QC: Quality control
RA: Rheumatoid arthritis
RNA-seq: RNA sequencing
SNP: Single nucleotide polymorphism
XCI: X chromosome inactivation
XMCPPAT: The Monte Carlo pedigree parental-asymmetry test on X chromosome
XPAT: The parental-asymmetry test on X chromosome
XPPAT: The pedigree parental-asymmetry test on X chromosome
