# Supplementary material for: A powerful parent-of-origin effects test for qualitative traits on X chromosome in general pedigrees
Source: BMC Bioinformatics. 2018 Jan 5;19:8. doi: 10.1186/s12859-017-2001-5 (PMC5756386; doi:10.1186/s12859-017-2001-5)
Supplement: Additional file 1 — Appendices and Supplementary figures. Appendix A Proof of E(SMC)=0 under the null hypothesis of no parent-of-origin effects; Appendix B Simulation study for the validity of XPPAT when testing parent-of-origin effects under X chromosome inactivation; Figs. A and B Power comparison of XPPATfull, XMCPPATt, XMCPPATfm, XPPAT and XPAT with N=150 and 300, respectively. The powers are calculated under four different parent-of-origin effect models with ρ=−0.05 based on 10,000 replicates at the significance level of 5%; Figs. C and D Power comparison of XPPATfull, XMCPPATt, XMCPPATfm, XPPAT and XPAT with N=150 and 300, respectively. The powers are calculated under four different parent-of-origin effect models with ρ=0.05 based on 10,000 replicates at the significance level of 5%. (PDF 72 kb) [file 12859_2017_2001_MOESM1_ESM.pdf]

Additional file 1

Appendices

Appendix A: Proof of $\mathbf{E}(S_{MC}) = 0$ under the null hypothesis of no parent-of-origin effects

For a single pedigree with n daughters (female nonfounders), let S_{MCj} be the contribution from daughter j to S_{MC} and B_j denote the event that daughter j is affected and heterozygous. Then, $S_{MC} = \sum_{j=1}^{n} S_{MCj}$ and

$$S_{MCj} = \sum_{G_m \in \mathbf{G}_m} I_{B_j} R_j(G_m, G_o)\Pr(G_m|G_o),$$

where $\mathbf{G}_m$ is the set of all possible genotypes for individuals with missing genotypes; I_{B_j} is an indicator function for B_j; $R_j = 1$ if the copies of allele D in the father of daughter j are more than or equal to those in the mother of daughter j, $R_j = -1$ if the copies of allele D in the father are less than those in the mother, and $R_j = 0$ otherwise, which depends on the genotypes (G_m, G_o) only. Let $\mathbf{G}_o$ be the set of all possible genotypes for individuals with known genotypes and $\mathbf{A}$ denote the set of all possible disease patterns for this pedigree. Note that $\Pr(G_m|G_o)$ may be different from $\Pr(G_m|G_o, A)$, where A is the collection of the phenotypes of all the individuals in the pedigree. So, when we treat A as random, we have

$$\begin{aligned}
\mathrm{E}(S_{MCj}) &= \sum_{A\in\mathbf{A}} \sum_{G_o\in\mathbf{G}_o} \sum_{G_m\in\mathbf{G}_m} I_{B_j} R_j(G_m, G_o)\Pr(G_m|G_o)\Pr(G_o|A)\Pr(A) \\
&= \sum_{A\in\mathbf{A}} \sum_{G_o\in\mathbf{G}_o} \sum_{G_m\in\mathbf{G}_m} I_{B_j} R_j(G_m, G_o)\Pr(G_m|G_o)\Pr(A|G_o)\Pr(G_o) \\
&= \sum_{G_o\in\mathbf{G}_o} \sum_{G_m\in\mathbf{G}_m} R_j(G_m, G_o)\Pr(G_m|G_o)\Pr(G_o) \sum_{A\in\mathbf{A}} I_{B_j}\Pr(A|G_o).
\end{aligned}$$

Note that

$$\sum_{A\in\mathbf{A}} I_{B_j}\Pr(A|G_o) = \begin{cases} \Pr(\text{daughter } j \text{ is affected}|C_j = 1) = a_j, & \text{if daughter } j \text{ is affected and heterozygous} \\ 0, & \text{otherwise} \end{cases},$$

where C_j denotes the count of allele D in daughter j. So, if daughter j is unaffected or homozygous, $\mathrm{E}(S_{MCj}) = 0$. On the other hand, if daughter j is affected and heterozygous,

under the null hypothesis of no parent-of-origin effects,

$$\begin{aligned}
\mathrm{E}(S_{MCj}) &= \sum_{G_o \in \mathbf{G}_o} \sum_{G_m \in \mathbf{G}_m} R_j(G_m, G_o)\mathrm{Pr}(G_m|G_o)\mathrm{Pr}(G_o)a_j \\
&= a_j \mathrm{E}(R_j) \\
&= 0,
\end{aligned}$$

where $\mathrm{E}(R_j) = 0$, because each heterozygous daughter has an equal chance of getting allele D either from her father or mother. Finally, we have

$$\begin{aligned}
\mathrm{E}(S_{MC}) &= \sum_{j=1}^{n} \mathrm{E}(S_{MCj}) \\
&= 0.
\end{aligned}$$

Appendix B: Simulation study for the validity of XPPAT when testing parent-of-origin effects under X chromosome inactivation

Imprinting effects and X chromosome inactivation (XCI) are two important biological mechanisms on X chromosome. XCI happens during early embryonic development in females whose paternal or maternal X chromosome is silenced to achieve dosage compensation between two sexes. It is generally a random process where both of the paternal and maternal X chromosomes have equal chance to be inactived. In this regard, XCI is easily confounded with imprinting effects. Here, we denote random XCI as XCI-R. However, recent studies have revealed that skewed XCI (XCI-S) is a biological plausibility, which has been defined as a significant deviation from XCI-R, for instance, the inactivation of one of the alleles in more than 75% of cells. In mice, XCI-S can be controlled by Xce gene or influenced by parent-of-origin effects. For human beings, XCI-S is more likely caused by secondary selection. The initial choice of active X chromosome is considered as random. During the body growth, when an X-linked mutation affects cells proliferation or survival, there will be a larger or smaller proportion of cells with the mutant allele active. For heterozygous females, positive selection cells with mutant allele will lead to more severe expression of the disease, whereas negative selection cells with mutant allele can provide protection from deleterious effects [1-2]. To investigate if our proposed methods are still valid for testing parent-of-origin effects under XCI-R and XCI-S, we conduct the following simulation study.

Consider a case-control design. For females, let $X = \{0, r, 2\}$ be the genotypic values for three unordered genotypes dd, Dd and DD at the candidate SNP locus on X chromosome, where $r \in [0, 2]$. For males, we use $X = \{0, s\}$ to denote the allelic values of alleles d and D, where $s \geq 0$. Let $Y = 1$ (0) denote that the individual (female or male) is affected (unaffected). Then, when there is no parent-of-origin effects, borrowing the idea of Wang et al. [3], the association between Y and X can be expressed using a logistic regression model

$$\text{Logit}(\Pr(Y = 1|X, z)) = \beta_0 + \beta X + \beta_z z, \quad (1)$$

where β_0 is the intercept; z is the gender of the individual with female and male being coded as 1 and 0, respectively, to indicate either female or male being at increased risk for disease; β and β_z are respectively the regression coefficient for X and that for z. In Wang et al. [3], s is set to be 2, which means dosage compensation (the effect of two risk alleles in females is equivalent to that of one-risk allele in males). According to Wang et al. [3], when $1 < r \leq 2$, this coding assumes a nonrandom XCI-S skewed toward to the disease allele D. For example, $r = 1.5$ indicates the XCI-S pattern in which 75% of the cells in a heterozygous female have the disease allele D active, whereas the other 25% of the cells have the normal allele d active.

Similarly, when $0 \leq r < 1$, this coding assumes a nonrandom XCI-S skewed toward to the normal allele d. In addition, $r = 1$ means XCI-R.

On the other hand, suppose that the genotype distribution in the control group and that in the case group of females follow trinomial distributions with probabilities (g_0, g_1, g_2) and (h_0, h_1, h_2), respectively, where g_0 (h_0), g_1 (h_1) and g_2 (h_2) are the genotype frequencies of dd, Dd and DD in the control (case) group, respectively. If the frequency p of allele D is given and Hardy-Weinberg equilibrium holds, then with $q = 1-p$, we have $g_0 = q^2$, $g_1 = 2pq$ and $g_2 = p^2$. Let $h_0/g_0 = a$, $h_1/g_1 = \lambda_{f1}(h_0/g_0) = \lambda_{f1}a$ and $h_2/g_2 = \lambda_{f2}(h_0/g_0) = \lambda_{f2}a$, where λ_{f1} and λ_{f2} are the odds ratios of genotypes Dd and DD compared to dd in females. As such, $\lambda_{f1} = \exp(\beta r)$ and $\lambda_{f2} = \exp(2\beta)$. By $h_0 + h_1 + h_2 = 1$, it is easy to show that $a = 1/[g_0 + g_1 \cdot \exp(\beta r) + g_2 \cdot \exp(2\beta)]$. Similarly, assume that the allele distribution in control group and that in the case group of males follow binomial distributions, and we use $\lambda_m = \exp(\beta s)$ to denote the odds ratio of allele D compared to d in males. According to Chen et al. [4], for females, $(\lambda_{f1} = \lambda_{f2} > 1)$, $(\lambda_{f1} = 1, \lambda_{f2} > 1)$ and $\lambda_{f1} = (\lambda_{f2} + 1)/2$ mean that the genetic models are dominant, recessive and additive, respectively. Further, when the association between the disease and X is present ($\beta \neq 0$), $\lambda_{f2} = \lambda_m$ (i.e., $s = 2$) is indicative of dosage compensation.

For simplicity, we only generate N parents-daughter trios under model (1), each with an affected daughter and her parents. For each family trio, we first generate the allele of the father according to the allele frequencies p and q and simulate the genotype of the mother based on the genotype distribution (g_0, g_1, g_2). Then, generate the genotype of the daughter from her parental genotypes. From Equation (1), we have the penetrances f_0, f_1 and f_2 for genotypes dd, Dd and DD as follows

$$f_0 = \frac{\exp(\beta_0 + \beta_z)}{1 + \exp(\beta_0 + \beta_z)}, \quad f_1 = \frac{\exp(\beta_0 + \beta r + \beta_z)}{1 + \exp(\beta_0 + \beta r + \beta_z)} \text{ and } f_2 = \frac{\exp(\beta_0 + 2\beta + \beta_z)}{1 + \exp(\beta_0 + 2\beta + \beta_z)}.$$

The affection status of the daughter is simulated based on the penetrances and her genotype. Here, N is taken to be 100 and 200. Then, we can calculate the value of the XPPAT test statistic based on these N parents-daughter trios. The frequency p of allele D is fixed at 0.1 and 0.3. We assume $\beta_0 = -2.55$ and $\beta_z = -0.0513$, and β takes values of 0.0953 and 0.2624. The true value of r is set to be 0, 0.5, 1, 1.5 and 2. We use the nominal significance levels $\alpha = 5\%$ and 1% for the type I error rate assessment. The simulation study is conducted based on 10,000 replications. Table A lists the corresponding empirical type I error rate of XPPAT. From the table, we find that XPPAT is still valid to test for imprinting effects on X chromosome under XCI, irrespective of XCI-R or XCI-S.

Finally, we would like to investigate the analogy and distinction between the simulation

of parent-of-origin effects on X chromosome and the simulation of XCI. For easy comparison, we only generate N parents-daughter trios, each with an affected daughter and her parents, in the simulation of parent-of-origin effects, just like the above-mentioned simulation of XCI. In the main text, we simulate the parent-of-origin effects (without XCI) by fixing the values of four penetrances f_{00}, f_{01}, f_{10} and f_{11} corresponding to genotypes d/d, d/D, D/d and D/D. In fact, we can also use the following logistic regression model to simulate the parent-of-origin effects

$$\text{Logit}(\Pr(Y = 1|X_1, X_2, z)) = \beta_0 + \beta_1 X_1 + \beta_2 X_2 + \beta_z z, \quad (2)$$

where (X_1, X_2) takes the values of $(0, 0)$, $(0, 1)$, $(1, 0)$ and $(1, 1)$ for the daughter with genotypes d/d, d/D, D/d and D/D, respectively; the regression coefficients β_1 and β_2 are respectively the genetic effect of maternally inherited allele D and that of paternally inherited allele D. From model (2), given the values of β_0, β_1, β_2 and β_z, we can get the corresponding penetrances f_{00}, f_{01}, f_{10} and f_{11} as follows

$$\begin{aligned} f_{00} &= \frac{\exp(\beta_0 + \beta_z)}{1 + \exp(\beta_0 + \beta_z)}, \quad f_{01} = \frac{\exp(\beta_0 + \beta_2 + \beta_z)}{1 + \exp(\beta_0 + \beta_2 + \beta_z)}, \\ f_{10} &= \frac{\exp(\beta_0 + \beta_1 + \beta_z)}{1 + \exp(\beta_0 + \beta_1 + \beta_z)} \quad \text{and } f_{11} = \frac{\exp(\beta_0 + \beta_1 + \beta_2 + \beta_z)}{1 + \exp(\beta_0 + \beta_1 + \beta_2 + \beta_z)}. \end{aligned}$$

Then, based on these penetrances, we can generate N parents-daughter trios in the way similar to XCI. Note that in model (2), $\beta_1 = \beta_2 = \beta$ implies $f_{01} = f_{10}$ (i.e., no parent-of-origin effects) and model (2) is reduced to be

$$\text{Logit}(\Pr(Y = 1|X^*, z)) = \beta_0 + \beta X^* + \beta_z z,$$

where $X^* = X_1 + X_2$ takes the values of 0, 1 and 2 for unordered genotypes dd, Dd and DD, respectively. Therefore, model (2) under no parent-of-origin effects is equivalent to model (1) under XCI-R.

References

1. Deng X, Berletch JB, Nguyen DK, Disteche CM. X chromosome regulation: diverse patterns in development, tissues and disease. Nat Rev Genet. 2014;15:367-78.
2. Medema RH, Boudewijn MT. The X factor: skewing X inactivation towards cancer. Cell. 2007;129:1253-4.
3. Wang J, Yu R, Shete S. X-chromosome genetic association test accounting for X-inactivation, skewed X-inactivation, and escape from X-inactivation. Genet Epidemiol. 2014;38:483–93.

4. Chen Z, Ng HKT, Li J, Liu Q, Huang H. Detecting associated single-nucleotide polymorphisms on the X chromosome in case control genome-wide association studies. Stat Methods Med Res. 2017;26:567-82.

Table A Empirical size (%) of XPPAT under X chromosome inactivation based on 10,000 replications

			$N = 100$		$N = 200$	
p	β	r	$\alpha = 5\%$	$\alpha = 1\%$	$\alpha = 5\%$	$\alpha = 1\%$
0.1	0.0953	0	4.67	0.86	5.19	1.15
	0.0953	0.5	5.15	1.03	5.15	1.02
	0.0953	1	5.43	0.84	5.00	0.95
	0.0953	1.5	5.44	0.88	5.36	1.05
	0.0953	2	5.51	0.92	5.08	0.97
	0.2624	0	5.06	0.78	5.11	1.10
	0.2624	0.5	5.49	0.93	4.83	0.86
	0.2624	1	5.42	0.70	4.83	0.98
	0.2624	1.5	5.36	0.83	4.96	0.94
	0.2624	2	5.23	1.06	4.65	0.85
0.3	0.0953	0	4.99	1.03	5.03	0.97
	0.0953	0.5	4.74	0.86	4.74	0.98
	0.0953	1	4.81	1.05	5.05	1.02
	0.0953	1.5	4.79	0.92	4.97	1.06
	0.0953	2	5.09	1.11	5.09	1.15
	0.2624	0	4.89	0.87	4.63	0.96
	0.2624	0.5	5.14	0.10	4.75	0.88
	0.2624	1	4.86	0.91	5.08	0.86
	0.2624	1.5	4.96	0.93	4.84	0.92
	0.2624	2	4.91	0.10	5.01	0.76

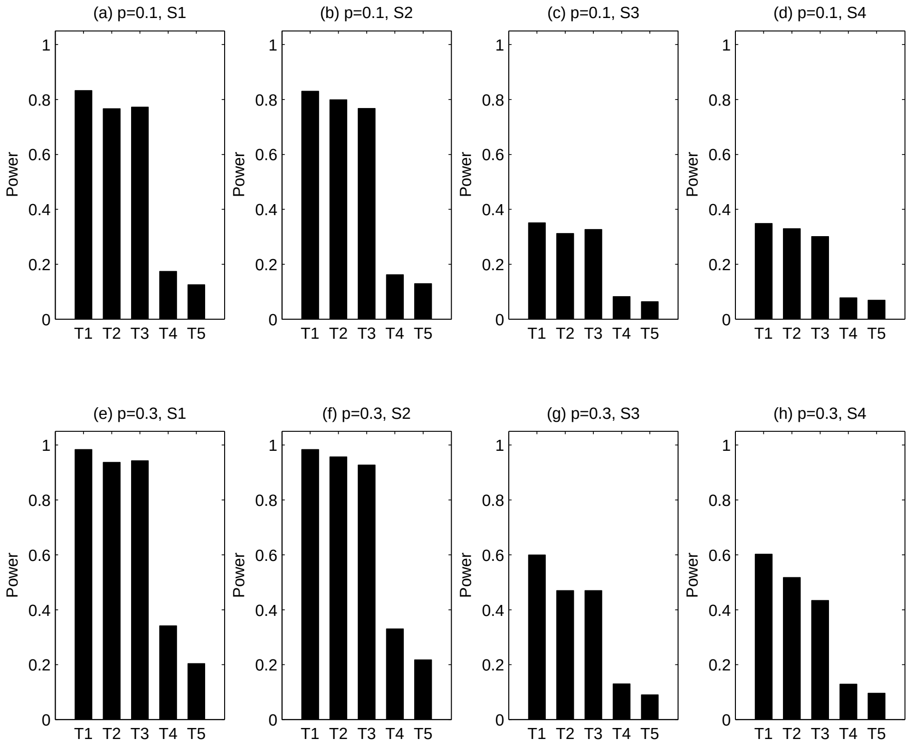

Fig. A Power comparison of T1: $\text{XPPAT}_{\text{full}}$, T2: $\text{XMCPPAT}_{\text{t}}$, T3: $\text{XMCPPAT}_{\text{fm}}$, T4: XPPAT and T5: XPAT. The powers are calculated under four different parent-of-origin effect models of S1: $(f_{11}, f_{10}, f_{01}, f_{00}) = (0.30, 0.30, 0.12, 0.12)$, S2: $(f_{11}, f_{10}, f_{01}, f_{00}) = (0.30, 0.12, 0.30, 0.12)$, S3: $(f_{11}, f_{10}, f_{01}, f_{00}) = (0.30, 0.26, 0.16, 0.12)$ and S4: $(f_{11}, f_{10}, f_{01}, f_{00})$ $=$ (0.30, 0.16, 0.26, 0.12) with $N = 150$ and $\rho = -0.05$ based on 10,000 replicates at the significance level of 5%. The first four tests are the proposed tests, while the last one is the existing test. The first row **(a)**, **(b)**, **(c)** and **(d)** with $p = 0.1$, while the second row **(e)**, **(f)**, **(g)** and **(h)** with $p = 0.3$.

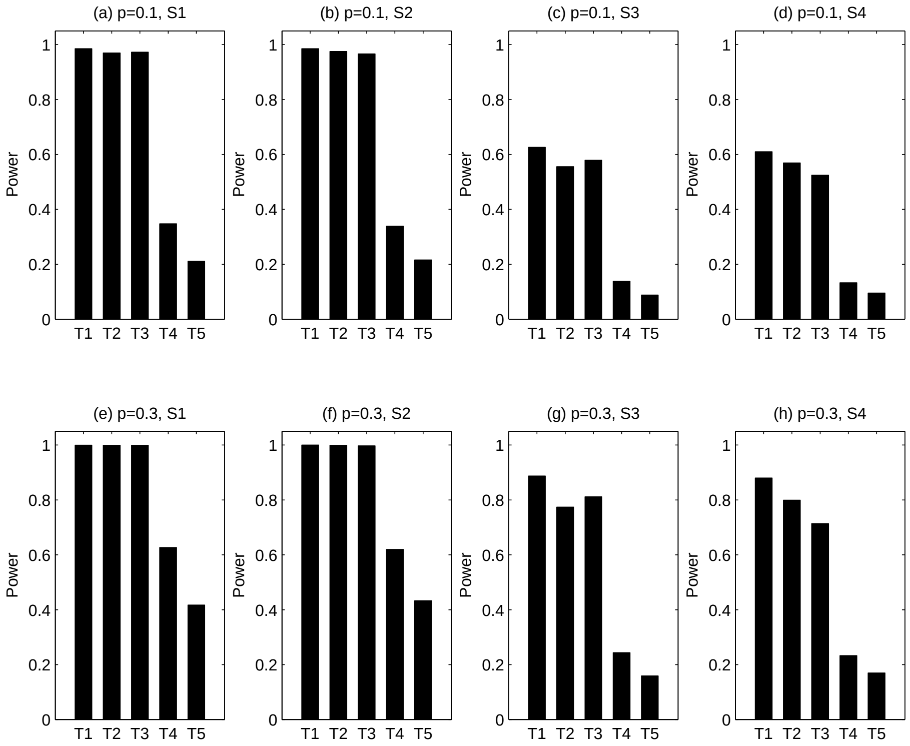

Fig. B Power comparison of T1: $\text{XPPAT}_{\text{full}}$, T2: $\text{XMCPPAT}_{\text{t}}$, T3: $\text{XMCPPAT}_{\text{fm}}$, T4: XPPAT and T5: XPAT. The powers are calculated under four different parent-of-origin effect models of S1: $(f_{11}, f_{10}, f_{01}, f_{00}) = (0.30, 0.30, 0.12, 0.12)$, S2: $(f_{11}, f_{10}, f_{01}, f_{00}) = (0.30, 0.12, 0.30, 0.12)$, S3: $(f_{11}, f_{10}, f_{01}, f_{00}) = (0.30, 0.26, 0.16, 0.12)$ and S4: $(f_{11}, f_{10}, f_{01}, f_{00}) = (0.30, 0.16, 0.26, 0.12)$ with $N = 300$ and $\rho = -0.05$ based on 10,000 replicates at the significance level of 5%. The first four tests are the proposed tests, while the last one is the existing test. The first row **(a)**, **(b)**, **(c)** and **(d)** with $p = 0.1$, while the second row **(e)**, **(f)**, **(g)** and **(h)** with $p = 0.3$.

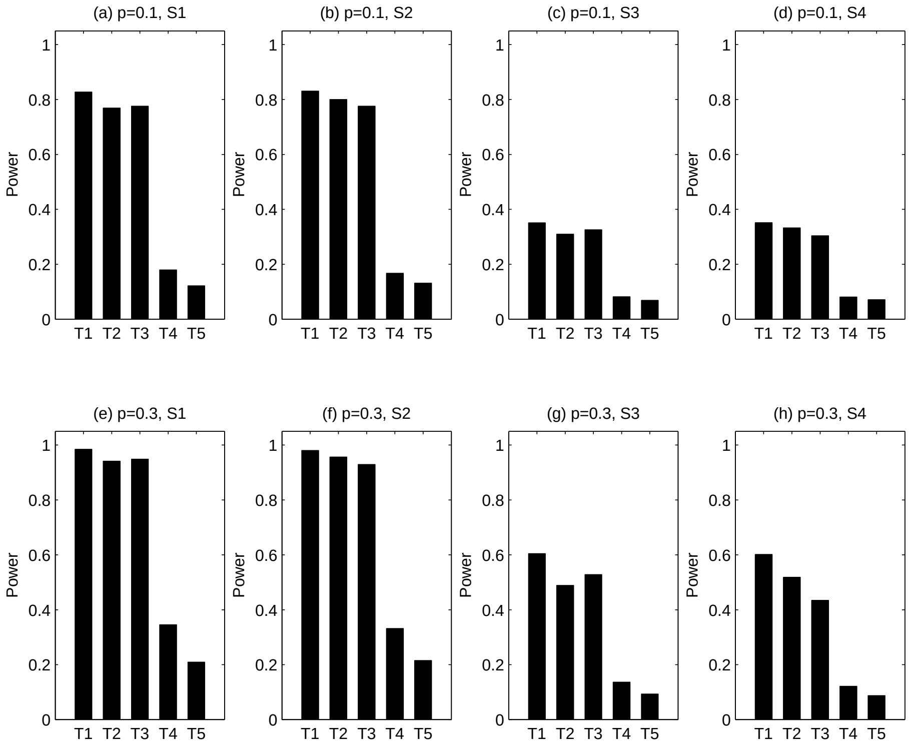

Fig. C Power comparison of T1: $\mathrm{XPPAT}_{\mathrm{full}}$, T2: $\mathrm{XMCPPAT}_{\mathrm{t}}$, T3: $\mathrm{XMCPPAT}_{\mathrm{fm}}$, T4: XPPAT and T5: XPAT. The powers are calculated under four different parent-of-origin effect models of S1: $(f_{11}, f_{10}, f_{01}, f_{00}) = (0.30, 0.30, 0.12, 0.12)$, S2: $(f_{11}, f_{10}, f_{01}, f_{00}) = (0.30, 0.12, 0.30, 0.12)$, S3: $(f_{11}, f_{10}, f_{01}, f_{00}) = (0.30, 0.26, 0.16, 0.12)$ and S4: $(f_{11}, f_{10}, f_{01}, f_{00})$ = (0.30, 0.16, 0.26, 0.12) with $N = 150$ and $\rho = 0.05$ based on 10,000 replicates at the significance level of 5%. The first four tests are the proposed tests, while the last one is the existing test. The first row **(a)**, **(b)**, **(c)** and **(d)** with $p = 0.1$, while the second row **(e)**, **(f)**, **(g)** and **(h)** with $p = 0.3$.

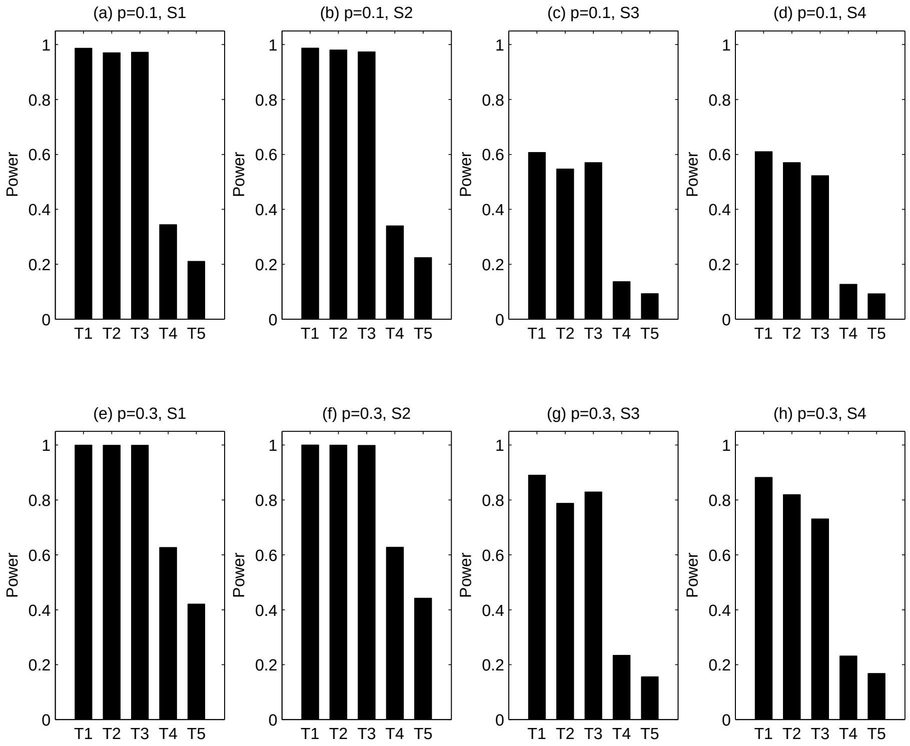

Fig. D Power comparison of T1: $\mathrm{XPPAT_{full}}$, T2: $\mathrm{XMCPPAT_{t}}$, T3: $\mathrm{XMCPPAT_{fm}}$, T4: XPPAT and T5: XPAT. The powers are calculated under four different parent-of-origin effect models of S1: $(f_{11}, f_{10}, f_{01}, f_{00}) = (0.30, 0.30, 0.12, 0.12)$, S2: $(f_{11}, f_{10}, f_{01}, f_{00}) = (0.30, 0.12, 0.30, 0.12)$, S3: $(f_{11}, f_{10}, f_{01}, f_{00}) = (0.30, 0.26, 0.16, 0.12)$ and S4: $(f_{11}, f_{10}, f_{01}, f_{00})$ = (0.30, 0.16, 0.26, 0.12) with $N = 300$ and $\rho = 0.05$ based on 10,000 replicates at the significance level of 5%. The first four tests are the proposed tests, while the last one is the existing test. The first row **(a)**, **(b)**, **(c)** and **(d)** with $p = 0.1$, while the second row **(e)**, **(f)**, **(g)** and **(h)** with $p = 0.3$.
